# Molecular and environmental analysis of Campania (Italy) sweet cherry (*Prunus avium* L.) cultivars for biocultural refugia identification and conservation

**DOI:** 10.1038/s41598-019-43226-2

**Published:** 2019-05-01

**Authors:** Livio Muccillo, Vittorio Colantuoni, Rosaria Sciarrillo, Giuseppe Baiamonte, Giovanni Salerno, Mario Marziano, Lina Sabatino, Carmine Guarino

**Affiliations:** Department of Sciences and Technologies, University of Sannio, Via Port’Arsa, 11, 82100 Benevento, Italy

**Keywords:** Haplotypes, Biodiversity

## Abstract

Conservation of agrobiodiversity is a major concern worldwide. Several strategies have been designed and programmed to reduce biodiversity erosion due to anthropic and non-anthropic causes. To this end, we set up a multidisciplinary approach based on the genetic analysis of selected cultivars and recognition of the environmental parameters. We genotyped the sweet cherry cultivars of Campania region in southern Italy by using simple sequence repeats and further investigated them by cluster analysis, disclosing a homogeneous genetic constitution, different from that of commercial accessions. By structure analysis we identified three distinct genetic clusters, each characterized by common and distinct alleles. Survey of the cultivars’ geographical distribution by quartic kernel function identified four preferred districts further characterized for soil origin, pedologic, agronomic features and urbanization impact. We correlated these environmental parameters, typical of the identified areas, with the three genetic pools and found a statistically significant association for each cluster. When we overlaid the cultivation traditions and cultural heritage, we found they have a dominant role; on these premises, we generated new territorial maps. In conclusion, we propose a novel methodological approach based on molecular, geo-pedological and cultural parameters with the aim to recognize biocultural refugia and preserve endangered or valuable cultivars.

## Introduction

Agro-biodiversity is characterized by the interplay of “natural resources” with the micro-environment, management and agricultural practices. Over the centuries, farmers have deliberately selected and preferentially cultivated certain cultivars due to their fruit qualities (genes/phenotypes) and their adaptation to specific habitats. The territories, where this selection was practiced, were generally confined and characterized by geomorphological and environmental features that guaranteed context specificity and characteristics so that a specific cultivar can be defined typical or autochthonous. The so-called “local varieties” are, therefore, a significant component of agro-biodiversity^1,2^.

Several authors^3,4^ have emphasized on traditional cultivars being one of the main sources of biodiversity, therefore the access to germplasm should be one of the pillars for sustainable agricultural development^1,5–7^. Traditional cultivars, however, have been reported to have a reduced, if any, market value and relegated to a marginal role; it could be argued, instead, that their absolute value is much higher than the sole monetary one^4^. In line with this reasoning, numerous international institutions and individual countries have developed a conservation-oriented strategy that involves the recognition, identification and conservation of traditional endangered varieties^8,9^. Their survey is a crucial goal to be pursued because loss of autochthonous cultivars is an indicator of biodiversity erosion that can lead to complete extinction of that variety and, ultimately, of the entire territory features^10^. In order to design appropriate conservation and management strategies, it is mandatory to acquire information on the genetic characteristics of the cultivars under investigation and relatedness among them^11–14^. This can be obtained through a specific “DNA fingerprinting” that can be used for traceability and for selecting diverse individuals with desired quality traits as breeding parents^6^. A molecular tool largely employed to detect such a genetic signature is represented by Simple Sequence Repeats (SSRs), recognized as useful genetic markers in plants and in animals due to their high degree of polymorphism, abundance in genomes, co-dominance and suitability for automation^15^. Conservation strategies, then, recognize, on one side, its absolute value in terms of biological and cultural heritage and, on the other, imply and justify its potential use in agricultural future programs^16,17^.

Campania Region is notoriously recognized as an area with a strong agricultural tradition starting from the Roman civilization times (the famous “Campania Felix”). Furthermore, Campania displays very diversified environmental and phytoclimatic characteristics that contribute to the formation of particular agro-pedo-environmental contexts so to permit specialized and selected cultivation traditions. It is clear, then, that the combination of such a diversity and traditions has produced over the centuries many specialties with a high degree of biodiversity. In Campania, sweet cherry (*Prunus avium* L.) is definitely a traditional culture, as evidenced by historical sources; its presence dates back to Pliny the Elder (I century A. C.), who described ten varieties and, since, it has always been a hallmark of the rural environment of this region^18^. Sweet cherry is a self-incompatible, out-breeding diploid species in the family of *Rosaceae*, having a genome of 2n = 16. Precise identification of the existing cultivars is essential for orchard establishment, efficient germplasm collection management and selection of genotypes in cherry breeding programs. In *Prunus*, microsatellites have been used for germplasm characterization^19^ and management^20^, determination of genetic diversity^21^, parentage analysis^22^, cultivar identification^23^ and mapping genetic linkage^24^. The growing demography and the continuous consumption of soil in many areas of the Campania region has reduced the natural agricultural locations with subsequent loss of certain varieties and traditional techniques of cultivation and conservation that were selected for a territorial vocation. In this paper, we analysed the genetic characteristics of several Campania sweet cherry cultivars and correlated these data with local territory features and urban settlements to generate a novel type of environmental map that could help in reducing the risk of extinction and preserving these habitats as “niche” or “biocultural refugia”^25–27^.

## Results and Discussion

### Genetic and molecular characterization of the cultivars

**Figure 1:**
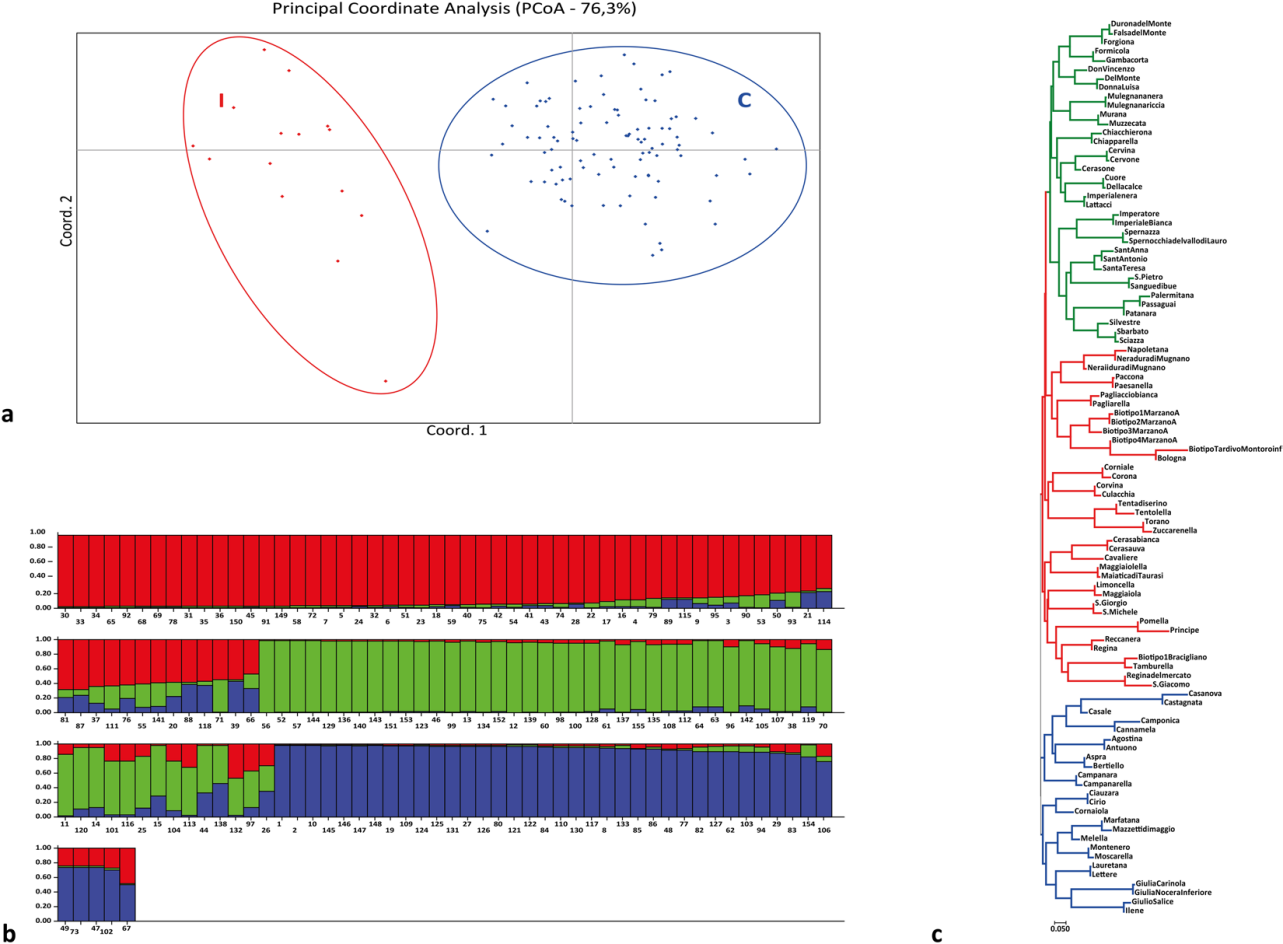
Genetic analysis of Campania sweet cherry cultivars. (**a**) Principal Coordinate Analysis (PCoA) of Campania’s sweet cherry cultivars generated by the tri-distance matrix of genetic euclidean similarity (76.3% of representation); (**b**) The hierarchial STRUCTURE analysis identified three genetic clusters by assignment probability of the genotypes of the 99 *Prunus avium* accessions. Each vertical bar corresponds to a distinct genotype and the proportion of its genome, q, assigned to the three clusters. Accession numbers refer to the cultivars listed in Supplementary Table S2. (**c**) Genetic relationships of Campania sweet cherry cultivars. The dendrogram was inferred using the Neighbor-Joining method^55^ and constructed using MEGA v7^56^. The optimal tree with the sum of branch length = 10.44 is shown.

The 113 cultivars of *P*. *avium* listed in Table 1 were genotyped by using the fifteen SSR primer pairs detailed in Materials and Methods. They have been shown to be very informative for fingerprinting, mapping and gene-flow studies in *P*. *avium*^28–30^; in addition, they generated easily scored markers and polymorphic amplification products for all the accessions analysed (Supplementary Tables S1–S3). We then carried out cluster analysis on the whole population to investigate the discrimination power of our SSR genetic markers and found an interesting differentiation by studying the tri-distance matrix of genetic Euclidean similarity by Principal Coordinates Analysis (PCoA) (Fig. 1a). Campania’s sweet cherries (cluster C for Campania) exhibit a remarkably different allelic pattern compared to commercial cultivars (including international ones and the new genotypes released from different cherry breeding programs) (cluster I for International). Furthermore, the international cultivars display a scattered spatial distribution with respect to the more grouped local ones, suggesting for the latter a homogeneous genetic constitution. This, however, does not rule out the existence of differences among them, as documented by the UPGMA (Unweighted Pair Group Method with Arithmetic mean) dendrogram reported in Supplementary Fig. S1.

**Table 1:** List of the ninety-nine sweet cherry cultivars sampled in the Campania region and, in bold and italic, the fourteen international cultivars used as reference.

Nr	Cultivar	Nr	Cultivar	Nr	Cultivar	Nr	Cultivar
1	Agostina	30	Forgiona	59	Pomella	88	Torano
2	Antuono	31	Giulio Salice	60	Recca nera	89	Agostegna
3	Aspra	32	Ilene	61	Regina	90	Selvatica Tardiva
4	Bertiello	33	Imperatore	62	Regina del mercato	91	Giulia Nocera Inferiore
5	Bologna	34	Imperiale nera	63	S.Giorgio	92	Giulia Carinola
6	Campanara	35	Lattacci	64	Sangue di bue	93	Imperiale Bianca
7	Campanarella	36	Lauretana	65	S.Michele	94	Palermitana
8	Camponica	37	Lettere	66	S.Pietro	95	Muzzecata
9	Cannamela	38	Limoncella	67	Sant’Anna	96	Biotipo 1Bracigliano.
10	Casale	39	Maggiaiola	68	Sant’Antonio	97	Biotipo 1 Marzano A.
11	Casanova	40	Maggiaiolella	69	Santa Teresa	98	Biotipo 2 Marzano A.
12	Castagnata	41	Maiatica di Taurasi	70	Sbarbato	98	Biotipo 3 Marzano A.
13	Cavaliere	42	Marfatana	71	Silvestre	98	Biotipo 4 Marzano A.
14	Cerasa bianca	43	Mazzetti di maggio	72	Spernocchia del Vallo di Lauro	99	Biotipo Tardivo Montoro Inferiore
15	Cerasauva	44	Melella	73	Tamburella	***100***	***Burlat C1***
16	Cerasone	45	Montenero	74	Tenta di Serino	***101***	***Compatto di Vignola***
17	Cervina	46	Moscarella	75	Zuccarenella	***102***	***Napoleon***
18	Cervone	47	Mulegnana nera	76	Principe	***103***	***Big Lory***
19	Chiacchierona	48	Mulegnana riccia	77	Corvina	***104***	***Durona di Cesena***
20	Chiapparella	49	Murana	78	Sciazza	***105***	***Early Star***
21	Ciauzara	50	Napoletana	79	Tentolella	***106***	***Giorgia***
22	Cirio	51	Nera dura di Mugnano	80	Gambacorta	***107***	***Hedelfingher***
23	Cornaiola	52	Nera ii dura di Mugnano	81	Formicola	***108***	***Lory Strong***
24	Corona	53	Paccona	82	Durona del Monte	***109***	***Malizia***
25	Culacchia	54	Paesanella	83	Falsa del Monte	***110***	***Negus***
26	Cuore	55	Pagliaccio bianca	84	Del Monte	***111***	***Somerset***
27	Della calce	56	Pagliarella	85	Spernazza	***112***	***Splendid***
28	Donna Luisa	57	Passaguai	86	S.Giacomo	***113***	***Synfonie***
29	Don Vincenzo	58	Patanara	87	Corniale

The analysis of the 155 Campania’s sweet cherry samples (N), belonging to the 99 putative autochthonous cultivars, identified 110 distinct alleles (Table 2). The allele average number (k) was 7.3 for each locus, ranging from 2 for EMPAs13 to a maximum of 14 for EMPA015, the most polymorphic marker. The number of alleles per locus varied according to the characteristics of the microsatellite repeats: it was low when the microsatellite repeat was simple and rather short and high with ascending repeat length or microsatellite complexity (Supplementary Table S1). The expected heterozygosity (He) for each individual locus ranged from 0.098 (EMPA011) to 0.804 (EMPAs06), with the mean being 0.617. The observed heterozygosity (Ho) ranged from 0.101 (EMPA011) to 0.895 (EMPAs02), with the mean being 0.630 indicating that the loci investigated have a high degree of discrimination. This result was confirmed by the Polymorphic Index Content (PIC) assayed within loci that ranged from 0.097 (EMPA011) to 0.775 (EMPAs06) with the majority of them (11 out of 15) exceeding the 0.5 threshold value. The Power of Discrimination (PD), whose values ranged from 0.250 (EMPA011) to 0.879 (EMPAs10), further supported these data. The mean Ho detected here (0.63) is similar to that reported for all outbreeding species, including almond (0.583 by Xie *et al*.)^31^ and plums (0.77 by Šiško)^32^ using equivalent SSR markers. A lower level of sweet cherry polymorphism has been detected by Stockinger *et al*.^33^ and Gerlach & Stoesser^34^ using RAPD markers and by Beaver *et al*.^35^ and Bošković & Tobutt^36^ using isozyme markers. This finding probably reflects: (*i*) a higher polymorphic sensitivity of the present SSRs compared to those reported in published works; (*ii*) a better outbreeding degree of the cultivars that is an index of the population structure analysed. In addition, we identified 26 private alleles (P.A.), 1.733 for each locus, with the private/total alleles richness ratio (ARR) that clearly indicated a high percentage of selective ability (from 18.2 to 44.4% of all alleles for each locus), except for EMPA001, EMPAs02, EMPAs13 and EMPAs01 that gave no private alleles. Finally, the estimated frequency of null alleles was close to zero for 12 out of 15 loci (only EMPA016, EMPA012 and EMPA015 exceeded the threshold value of |0.10|), with some of them slightly negative (negative values imply an excess of observed heterozygote genotypes). Furthermore, a locus with a large positive estimate of null allele frequency indicates an excess of homozygotes but does not necessarily imply the presence of a null allele, as shown for EMPA015 (0.289). These results indicate that the loci identified and the primers used have a high discrimination power for each cultivar within the entire population. Finally, we analysed whether our population is in a Hardy-Weinberg equilibrium by calculating the Inbreeding coefficient (Fis) and the Fixation Index (Fst). Fis negative values reflect high heterozygosity and a Fixation Index (Fst) above 0.25 for all loci, indicate genetic differentiation among subpopulations. Moreover, Fit (Fitness), indicates heterozygosity reduction for each individual and ranges from −1 to +1, with the highest value corresponding to the lowest heterozygosity within the entire population. In our case, Fit was close to 0 for 12 out of 15 loci.

**Table 2:** Statistical analysis’ data obtained by genotyping the Campania’s sweet cherry cultivars using the fifteen SSR microsatellite markers.

Locus	k	N	He	Ho	PIC	PD	P. A.	ARR	F(Null)	Fis	Fst	Fit
EMPA001	7	151	0.692	0.669	0.642	0.841	0	ND	0.0152	−0.739	0.433	0.014
EMPA004	7	154	0.679	0.662	0.621	0.826	2	0,286	0.0208	−0.770	0.479	0.079
EMPA005	9	149	0.586	0.557	0.538	0.775	3	0,333	0.0063	−0.727	0.519	0.170
EMPA011	9	149	0.098	0.101	0.097	0.250	4	0,444	−0.0161	−0.754	0.713	0.496
EMPA018	10	153	0.681	0.765	0.625	0.777	3	0,300	−0.0695	−0.808	0.372	−0.135
EMPA015	14	147	0.738	0.415	0.7	0.857	5	0,357	0.2898	−0.585	0.703	0.529
EMPA012	4	152	0.417	0.309	0.34	0.599	1	0,250	0.1435	−0.601	0.549	0.278
EMPAs06	11	154	0.804	0.74	0.775	0.929	2	0,182	0.0388	−0.746	0.467	0.069
EMPAs02	6	153	0.76	0.895	0.72	0.866	0	ND	−0.0935	−0.837	0.373	−0.152
EMPAs10	9	155	0.76	0.819	0.721	0.879	2	0,222	−0.0438	−0.814	0.411	−0.068
EMPA016	5	151	0.606	0.808	0.524	0.666	1	0,200	−0.1508	−0.865	0.320	−0.268
EMPAs13	2	155	0.49	0.548	0.369	0.803	0	ND	−0.0583	−0.791	0.419	−0.041
EMPAs12	8	153	0.704	0.83	0.662	0.854	2	0,200	−0.0928	−0.814	0.369	−0.144
EMPAs01	5	153	0.718	0.843	0.662	0.803	0	ND	−0.0853	−0.844	0.391	−0.123
EMPAs14	4	154	0.526	0.494	0.412	0.677	1	0,250	0.032	−0.734	0.468	0.078
Mean	**7**.**333**	**152**.**2**	**0**.**617**	**0**.**630**	**0**.**561**	**0**.**760**	**1**.**733**	**0**.**275**	−0.004	**−0**.**762**	**0**.**466**	**0**.**052**

We genotyped 155 Campania sweet cherry individuals corresponding to 99 cultivars, using the fifteen SSR microsatellite markers. For each locus, the allele number (k), the number of amplified samples (N), the expected (He) and observed (Ho) heterozygosity, the Polymorphic Index Content (PIC), Power Discrimination (PD), the private (P.A.)/total alleles richness ratio (ARR), null alleles frequencies F (Null), Inbreeding coefficient (Fis), Fixation Index (Fst) and Fit (Fitness) were calculated.

### Cluster analysis

We then used STRUCTURE analysis to infer the real number of genetic clusters in *P*. *avium* (true K) and subsequently evaluate it as reported by Evanno *et al*.^37^. This model assumes that unknown K ancestral populations are at both Hardy-Weinberg and linkage equilibrium^38^ and, nonetheless, all individuals are probabilistically assigned to clusters or connected to each of several clusters if their genotypes are mixed. Under the admixture model of genetic rearrangements, an individual’s probability of assignment to each cluster (q) can be interpreted as the proportion of that individual’s genome that originated from each cluster (Fig. 1b)^39^. When the program was first run, with a burning period from 5000 to 50000, i.e. in less stringent conditions, a maximum in log likelihood was reached at the second run; when the program was run with a burning period from 500000 to 750000, i. e. with a higher permutation level, the maximum was reached at the third run (Supplementary Fig. S2). The difference in the two Ks suggests that the value obtained in the latter burning is better aligned with the genetic constitution of our populations and, thus, allows to identify three distinct genetic clusters. Assignment probability of the genotypes of the 99 accessions to the three genetic pools was also illustrated by hierarchical STRUCTURE analysis (Fig. 1b). Each vertical bar corresponds to a distinct genotype and the proportion of its genome, q, assigned to the three clusters.

**Figure 2:**
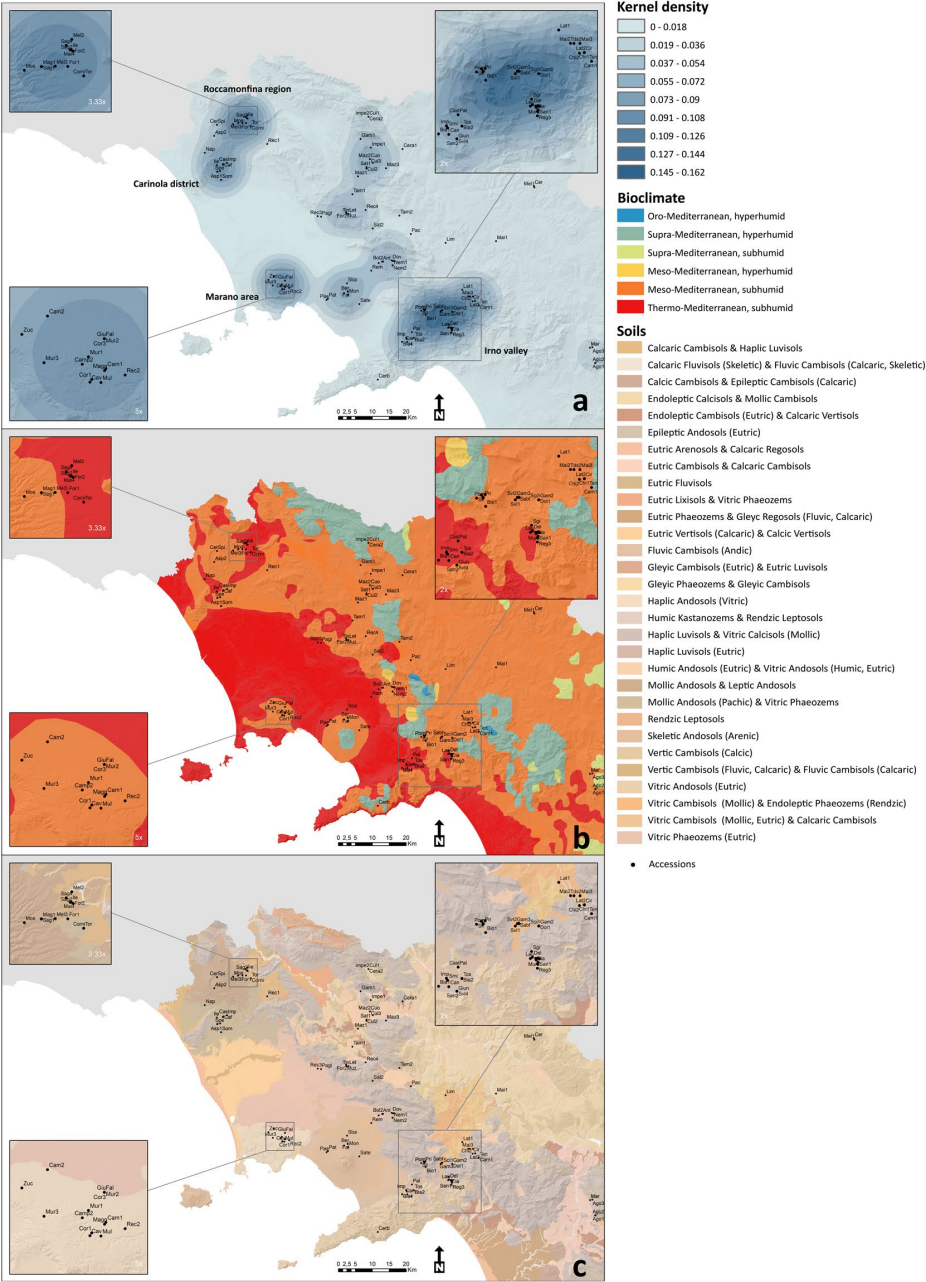
Sweet cherry Campania’s accessions were examined for (**a**) geographical distribution through kernel density analysis, (**b**) bioclimate and (**c**) soil composition.

We analysed our population also with the Neighbour Joining method and recognized three major related groups, strongly supporting the results obtained with the STRUCTURE analysis as shown by the dendrogram in Fig. 2c. This survey also identified 64 cases of homonymia [genetic distance rate (gdr) <5%]:

1. Cluster 1: Ilene/Giulio Salice, Giulia(Nocera Inferiore)/Giulia (Carinola), Lettere/Lauretana, Moscarella/Montenero, Mazzetti di Maggio/Marfatana, Cirio/Ciauzara, Campanarella/Campanara, Bertiello/Aspra, Antuono/Agostina; Cluster 2: San Michele/San Giorgio, Maggiaiola/Limoncella, Maiatica di Taurasi/Maggiaiolella, Cerasa Uva/Cerasa bianca, Zuccarenella/Torano, Culacchia/Corvina, Corona/Corniale, the two Biotipo (1/2), Pagliaccio Bianca/Pagliarella, Paccona/Paesanella, Napoletana/ Nera dura di Mugnano; Cluster 3: Sciazza/Sbarbato, Palermitana/Passaguai, San Pietro/Sangue di bue, Spernazza/Spernocchia del vallo di Lauro, Imperatore/Imperiale bianca, Imperiale nera/Lattacci, Cuore/Della calce, Cervina/Cervone, Murana/Muzzecata, Mulegnana nera/Mulegnana riccia, Del Monte/Donna Luisa, Durona del Monte/Falsa del Monte. The three clusters account for 54% of total Campania’s sweet cherry cultivars.
2. The genotypes of 12 cultivars account for 10.5%, suggesting they are clones derived from an ancestral variety [5% < genetic distance rate (gdr) <10%], Tentolella/Tenta di Serino, Biotipo 3 belongs to the other two; Sant’Anna/ Sant’Antonio with Santa Teresa; Chiacchierona/Chiapparella; Don Vincenzo with Del Monte/Donna Luisa, Formicola/Gambacorta.
3. The remaining cultivars, not listed above, exhibit a unique identity and account for 35.5% of total accessions.

All together these data indicate that we can identify three distinct genetic clusters among the *P*. *avium* cultivars investigated here. They represent true genetic structures as deduced by the presence of highly assigned individuals to each cluster and by the biologically meaningful clustering of individuals within accessions. By applying different methodological approaches, we can assign the 99 sweet cherry varieties analysed to three distinct gene pools, each characterized by distinct and common genetic traits.

### Geographical distribution, soil constitution and environmental characteristics of the cultivation hotspots of the investigated cultivars

Thereafter, we assessed the cultivars’ spatial density using a quartic kernel function to calculate the density of sampling point features. This analysis highlighted four hotspots: the Marano area, the Roccamonfina region (Marzano Appio, in particular), the Carinola district and the Irno valley (Fig. 2a). We also overlaid the cultivars locations with bioclimate and found that 100% of samples are located in the subhumid hombrotype that appears to be the best fitted for sweet cherry cultivation. Indeed, 83.5% of samples are exposed to the Meso-Mediterranean and the remaining 16.5% to the Thermo-Mediterranean bioclimate indicating that it cannot be considered a discriminating factor among the investigated cultivars (Fig. 2b and Supplementary Fig. S3).

Each of the four hotspots was further analysed for the soil origin according to the World Reference Base international standard for soil classification system endorsed by the International Union of Soil Sciences (Fig. 2c). We found that the Marano and Roccamonfina areas have a substantial and primary volcanic cover. Andosols are prevalent and originate from a volcanic substrate, pyroclastic/volcanoclastic in particular, made of ashes, sands and lapilli, due to fall and flow phenomena originating from significant explosive eruptions in the “Campi Flegrei” area rather than from the currently inactive Roccamonfina volcano. The Carinola and Roccamonfina areas, in addition, exhibit a unique mollic character where volcanic or volcano-clastic soils develop on a soft cineritic substrate, easily alterable, producing clays. The Irno valley, instead, is made of alluvial deposits mixed with volcano-clastites and pyro-clastites in their primary arrangement. In the identified hotspots, all types of soils are well developed, rich in organic matter; therefore, they are fertile, retain moisture efficiently and can be easily worked (Fig. 2c).

**Figure 3:**
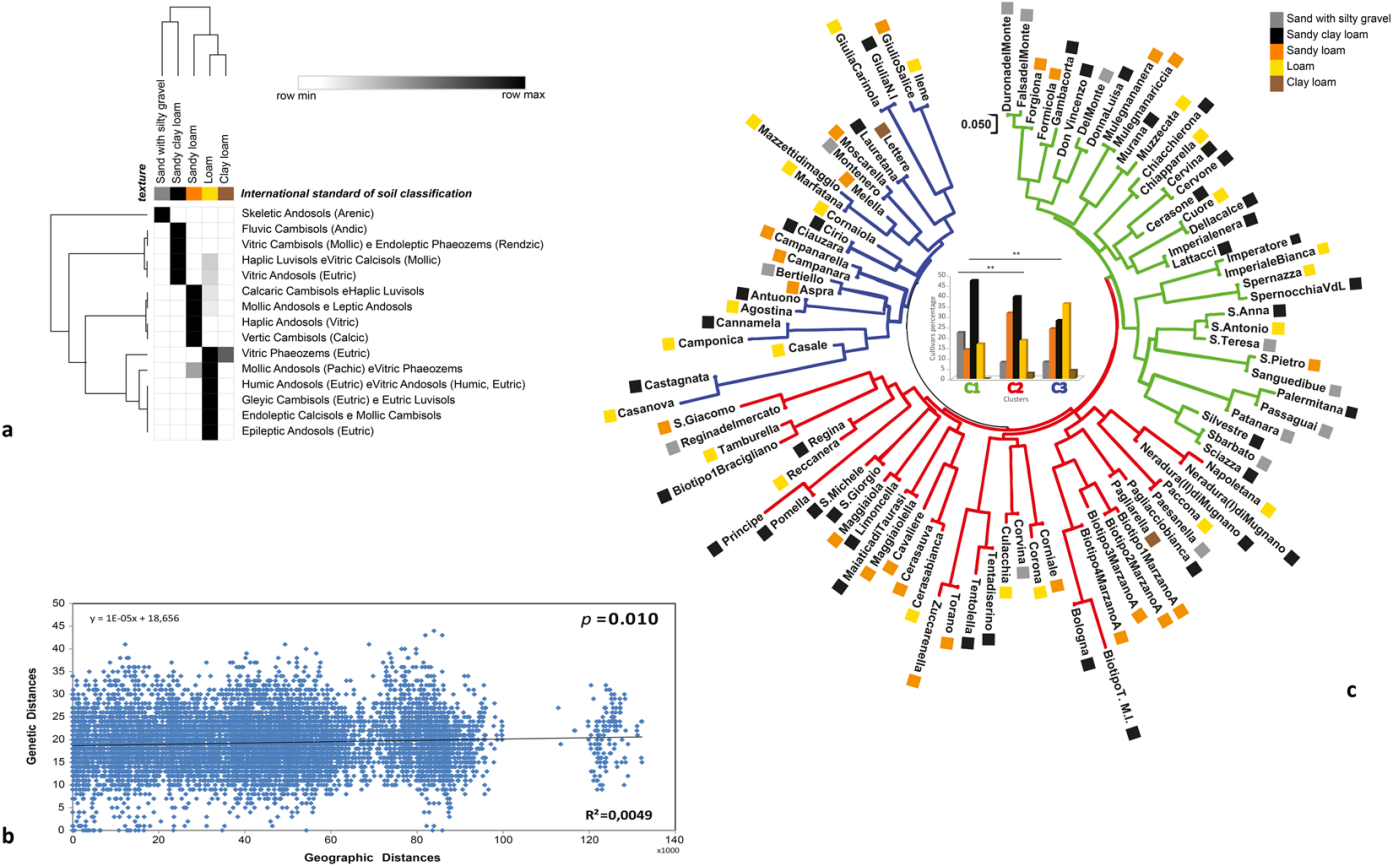
Correlation of the genetic constitution of the sweet cherry cultivars with geo-pedologic and soil texture composition. (**a**) The heatmap correlates sweet cherries’ soil origin, as in Fig. 2c, with the relative soil texture. The five types of textures identified (Sand with silty gravel, grey, Sandy clay loam, black, Sandy loam, orange, Loam, yellow, Clay loam, brown) are represented; (**b**) Mantel test draws the overlapping Genetic vs Geographic distances (*p* < 0.01, R^2^ = 0.0049); (**c**) The three genetic clusters identified were associated with the texture features, as in (**a**). The dendrogram was inferred using the Neighbor-Joining method^55^ and each cultivar associated with the preferred soil texture, as illustrated in the histograms in the centre, **p < 0.001.

We then analysed pedologic, agronomic and ecomosaic parameters as the major differences among the four hotspots can be referred to the top soils, i.e. those interested in roots exploration. These layers, indeed, significantly differ since they heavily depend on human activities (cultivation methods, nutritional inputs), but also on soil’s physical structure or texture. This, in fact, affects cherry trees distribution as it strongly influences soil hydrology (water holding capacity, infiltration, percolation) and possibly guides selection of specific types of trees to place in orchards. The cherry trees prefer sunny, damp clayey soils, without water stagnation and, in fact, in typical cherry districts, the cation-exchange capacity is primarily due to the presence of pyroclastic soils and high amount of organic matter, factors that improve the agricultural quality of cherry tree soils. More specifically, the Carinola district soil, as part of internal volcanic plains, is moderately deep, with medium texture that ensures good permeability, a typical requirement for sweet cherry cultivation. The soil pH is slightly to moderately alkaline, with a good fertility due to cation-exchange capacity and to the high base-cation saturation ratio, ensuring a good amount of K, Ca and Mg adsorbed on colloids (Table 3). The ecomosaic of this district has a plant covering that consists of specialized orchards (90%) with very few residual nuclei of autochthonous woods with *Quercus pubescens* Willd prevailing. The Roccamonfina region’s soil is part of the volcano’s caldera, includes the flat pedestrian areas and is deep with a moderately coarse texture without limestone. The land is typically used for fruit crops. The ecomosaic is very interesting because specialized cherry and chestnut trees orchards are interspersed with chestnut coppices and mesophilous Mediterranean woods with prevalence of *Quercus pubescens* Willd and *Quecus ilex* L. The Marano (and Chiaiano) district’s soil originates from the volcanic system of Campi Flegrei, is very deep with good permeability, has a moderate coarse texture and a fairly low pH. Limestone is absent. The ecomosaic is dramatically modified due to a massive urbanization from the city of Naples and restricted to slope lands towards Marano and Chiaiano where chestnut coppices are interspersed with mixed orchards with a prevalence of very valuable cherry cultivars. Because of the traditional and economic value of these orchards, the area is protected within the metropolitan park. Finally, the Irno valley’s soil is deep with medium to moderate fine texture, moderately alkaline pH, non-calcareous, with high base-cation saturation ratio (Table 3). Also in this location the ecomosaic is affected by a strong urbanization that left small areas dedicated to specialized mixed orchards and gardens. Due to the landscape of floodplains, the valley has a distinct vocation towards cherry tree cultivation subjected to conservation and protection. The heatmap in Fig. 3a illustrates the correlations of the different texture characteristics with those derived from the international soil classification system and recapitulates the cherry trees distribution in the four identified Campania’s hotspots. A detailed description of the pedologic, agronomic and ecomosaic parameters is reported in Table 3.

**Table 3:** Chemical-physical characteristics of the identified Campania’s sweet cherry hotspots top-soils.

Location	Depth (cm)	Texture	pH	Cation-exchange capacity (meq/100 g)	Organic matter (g/kg)
Carinola	100	Loam	7.6	22.7	18
Roccamonfina	150+	clay loam to sandy clay loam	5.8	20.5	21
Marano - Chiaiano	150+	Coarse sandy loam	5.8	18.4	16
Valle dell’Irno	150+	Loam to Sandy Clay Loam	7.9	28.1	12

The four hotspots location, depth (cm), texture, pH, cation-exchange capacity (meq/100 g), organic matter (g/Kg) are reported.

### Relationship between the genetic constitution of the cultivars, their geographic distribution and soil-pedologic characteristics

Lastly, we attempted to correlate individuals’ genetic inter-distances matrix (Euclidean) with the corresponding geographical distribution (latitude/longitude) by performing a Mantel Test using GenAlEx 6.41 software^40^. We found a positive and significant correlation between geographical and genetic distances (R^2^ = 0.0049, *p* = 0.01) (Fig. 3b) indicating that the three sweet cherry genetic clusters identified above exhibit a preferential geographical distribution over distinct areas of the Campania region, likely due to their unique characteristics. Despite the high values of biodiversity’s features still present, the low R^2^ and the scatter-plot pattern suggest that geographical/anthropic issues could reduce the spreading as gene flow that likely occurs among neighbouring populations, underscoring a starting genetic erosion. This association may be attributed to the fact that sweet cherry genetic diversity, as for most fruit woodies, is influenced by the adaptation to climatic and environmental conditions (both in biotic and abiotic terms) as well as by anthropic factors, critical parameters for their propagation. Indeed, sweet cherries of Cluster 1 (C1) are preferentially distributed and cultivated over a soil with volcanic sandy clay loam (black) or enriched on sand with silty gravel (gray). Sweet cherries of Cluster 2 (C2) are also distributed over a soil of volcanic origin (volcanic sandy clay loam, black, and volcanic sandy loam (orange). The optimal layer of sweet cherries Cluster 3 (C3) is, instead, characterized by loam soils (see Fig. 3c). This type of distribution supports the hypothesis of a stringent relationship of the genetic/molecular signature with the environment. These characteristics also preserve the sweet cherry cultivars from the growing demographic expansion that endangers agro-biodiversity, as witnessed by the *ad hoc* decrees issued by Regione Campania and Neaples metropolitan city authority to protect and preserve some typical cultivation areas from further urban settlements.

Another result of this latter correlation is that the sweet cherry orchards are located into urban or suburban areas with prevalent human activities that date back to the Greek and Roman civilization times. The Marano area corresponds to “Campi Flegrei” where the Greek immigrants landed in Italy and settled their first locations. It also corresponds to where the cities of Cuma and Puteoli flourished during the Roman times, likely because of the fertile land and good climate. The Roccamonfina volcanic area was the place where the pre-Roman inhabitants settled their communities, presumably for the same reasons as for the Irno valley, which was colonized because of soil fertility, proximity to water and gentle climate. Of note, these initial settings persisted over the centuries because agriculture has been the main source of food and income. Even today it is still possible to relate selected and typical cultivars with well-identified areas that, with good reason, can be defined “niches” or “biocultural refugia”^41–43^. In fact, the chemical-physical properties, the taste and flavour of these cultivars, their ability to resist to pathogens and productivity in terms of crop yield and farmers’ income are unique to the identified areas and cannot be or can be only partially reproduced in other contexts. A typical example is represented by the famous red pulp varieties (i.e. Mulegnana nera and riccia from Marano-Chiaiano area and Giulio Salice clones cultivated in the Carinola area) rich in anthocyanidines, likely because of their own substrates specificities.

Finally, we took into account the cultural heritage and cultivation traditions of the identified hotspots and found they play a crucial role in the cultivars’ selection so to overwhelm many of other features. These considerations imply that behind the choice of a selected cultivar there are genetic, bioclimatic, geo-pedological, cultural and traditional cultivation factors. We overlaid all these data to generate a new type of territorial map that provides sufficient information density at the scale of interest with the advantage of having unit boundaries based on the mentioned criteria and not solely on administrative ones as were the thematic maps of the Regional Territorial Plan (PTR).

Collectively, these data indicate that the genetic constitution of the Campania sweet cherry cultivars analysed correlates not only with the spatial and geographical distribution, with the soil and pedologic features but also with the cultural heritage of the cultivation areas.

## Conclusions

In this study, we employed a multidisciplinary approach that combines molecular/genetic, environmental and geo-pedological parameters to characterize the sweet cherry populations of the Campania region located in Southern Italy. We chose this region because there is a long-lasting tradition of sweet cherry cultivation that dates back to the Roman times and because, as from the eighteen century, some areas were dedicated uniquely or mostly to this crop. Survey of the genetic signature of the local varieties identified three major groups, each comprising cultivars characterized by common and domestic genetic features. Only few studies have been carried out so far to analyse the genetic constitution and structure of sweet cherry populations in general and of the Campania’s cultivars in particular. Our data indicate that these cultivars show a defined and wide genetic differentiation as compared with reference cultivars. The existence of genetic diversity in a given population underlines the plasticity of the genetic material and its ability to best accommodate to the environmental conditions and to reprogram the metabolism to local available resources. Moreover, the SSR markers employed result effective in differentiating cultivars and the data are consistent with those previously and independently reported^28–30^.

The three genetic clusters identified positively correlate with spatial distribution and soil, geo-pedologic and environmental parameters. Interestingly, each gene pool exhibits a preferential distribution over the major hotspots identified by the spatial density analysis. Indeed, two areas (Marano and Roccamonfina) have a volcanic origin and share some common features as one derives from the Campi Flegrei caldera and the other from the more distinct and distant Roccamonfina region, likely generated from a different volcano. While gene clusters 1 and 2 are preferentially distributed over these two areas, cluster 3 appears to have as site of election the Irno valley that has a completely different soil and environmental composition. The cultivation traditions of the identified areas originating from different cultural backgrounds provide a higher level of complexity and, for some aspects, overwhelm other considered parameters.

Taking into account all these criteria, we generated a new map based on genetic/molecular, ecological and environmental data and not only or exclusively on soil composition or administrative criteria as most of those presently available. Our map may be a powerful tool for conservation and management strategies to preserve biodiversity, a topic that is acquiring growing interest as it is constantly endangered or eroded by anthropic and non-anthropic causes. As a result, we may lose useful and critical traits, as those related to resistance to parasites, tolerance to extreme temperature or to complete or partial water loss, the first steps towards complete extinction of that population. Our map may also help to stimulate specific productions in selected areas to ensure their quality and characteristics and may be a support for new strategies to select distinct areas as “niches” or “biocultural refugia”. The derived products combine genetic backgrounds with specific neighborhoods, support the sustainability of certain cultivars and provide an added value on the market.

## Methods

### DNA isolation and amplification

Genomic DNA was extracted from 80 ng of young leaf tissue with a cetytrimethylammonium bromide based method^44^ and genotyped with a set of fifteen microsatellite primers pairs developed from *P*. *avium*. Eight primer sets were isolated by enrichment from *P*. *avium* “Napoleon”^29^ and seven identified from a *P*. *avium* “Charger” genomic DNA library^28^. The selected microsatellites map to seven of the eight linkage groups of the *P*. *avium* “Napoleon” x *Prunus nipponica* map as reported in Supplementary Table S1^45^. Fifty ng of each template DNA was subsequently subjected to PCR amplification in a total volume of 20 µl containing 0.2 mmol/L dNTPs, 10 mmol/L Tris–HCl (pH 9.0), 0.3 Units of AmpliTaq Gold PCR Mastermix (Life Technologies, Thermo Fisher, Foster City, CA, USA), 0.3 µmol µl each primer. The PCR conditions were: 95 °C for 5 min followed by 40 cycles of 95 °C for 50 s, annealing temperature for 1 min, 72 °C for 50 s, and a final cycle of 72 °C for 7 min. All forward primers were labeled with the fluorescent dyes (VIC, 6-FAM, NED and PET) (Life Technologies, Thermo Fisher, Foster City, CA, USA) and PCR products analyzed using an ABI 3100 genetic analyser (Applied Biosystems, Thermo Fisher, CA, USA) following the manufacturer’s instructions; GeneScan 500-ROX (Life Technologies, Thermo Fisher, Foster City, CA, USA) was used as internal size standard. Data were collected and allele size determined using the Peak Scanner™ v.1.2 software (Life Technologies, Thermo Fisher, Foster City, CA, USA).

### Data Analysis

Allele numbers, expected (He) and observed (Ho) heterozigosity, Polymorphic Index Content (PIC), and frequency of null alleles (F-null) were calculated using the CERVUS 3.0.3 software (http://www.fieldgenetics.com/pages/home.jsp, Tristan Marshall 1998–2007). A model-based Bayesian clustering method, implemented in the program STRUCTURE version 2.3.4^38^, was used to elucidate the genetic variations and identify the number of genetically distinct gene clusters within *P*. *avium*. The program STRUCTURE identifies K clusters of individuals based on the multiloci genotypes of all sampled accessions. STRUCTURE was run twice on the total set of 155 distinct genotypes: in the first run, with a burning period from 5*10^3^ to 5*10^4^ Markov chain Monte Carlo replications, the ΔK ranged between 1 and 25; in the second run, with a burning period from 5*10^5^ to 7.5*10^5^ Markov chain Monte Carlo replications, the ΔK ranged between 1 and 10. The admixture model, which aestimates the fraction of ancestry from each cluster for each individual, was used and the analyses run with the correlated allele frequencies^46^. The appropriate K value was chosen as reported by Evanno *et al*.^37^. The ∆K formula is given by the following:

$${\rm{\Delta }}{\rm{K}}=[{\rm{L}}^{\prime\prime} ({\rm{K}})]/{\rm{St}}.{\rm{Dev}};$$

where

$${\rm{L}}^{\prime\prime} ({\rm{K}})={\rm{L}}^{\prime} ({\rm{K}}){\rm{n}}\mbox{--}{\rm{L}}^{\prime} ({\rm{K}}){\rm{n}}\mbox{--}{\rm{1}};$$

and

$${\rm{L}}^{\prime} ({\rm{K}})=L(K){\rm{n}}\mbox{--}{\rm{L}}({\rm{K}}){\rm{n}}\mbox{--}{\rm{1}}.$$

L(K) is the average of n values of Ln P(D), that is the estimation of data log likehood, given the current values of P (Probability of inferred n run) in relation to Q (matrix of corresponding P values for run n).

Power of Discrimination (PD) was calculated as described by Kloosterman *et al*.^47^. Allele Richness Ratio (ARR) was evaluated dividing Private Alleles (P.A.) by Total Alleles (T.A.) for each locus. The genetic relationships among genotypes, Fis (Inbreeding Coefficient), Fit (Fitness) and Fst (Fixation Index) were estimated by the Nei index^48^ using the GenAlEx 6.41 software^40^, [http://www.anu.edu.au/BoZo/GenAlEx/]. A dendrogram was generated based on the neighboring joining NJ, using MEGA v.7 software through which a genetic distance matrix was obtained^49^.

### Agricultural investigation and samples material harvesting

Campania Region was divided into homogeneous areas that best represent the main environmental and landscape characteristics of the different territories, as defined by the environmental and agroforestry thematic maps contained in the Regional Territorial Plan (PTR) [Law. n. 13/2008; Corine Land Cover CLC 16-4/2012 version. 2006; Regione Campania 2006]. In particular, twenty-eight Campania Rural Territorial Systems were identified upon assembling reasonably homogeneous municipal territories according to:

- phytological and pedological aspects that affect production’s potentials;
- dominant agricultural and forestry uses;
- forms and structures of the agricultural landscape and their evolution during the last fifty years;
- relations with the urban and infrastructural system.

The resulting rural systems tend to correspond, on a regional scale, to the main emerging ecogeographic and landscape units, such as Matese, Piana Campana, Volcanic System of Somma-Vesuvio, Cilento Costiera and others, even though the unit boundaries were defined on administrative rather than ecological criteria^50^.

The sweet cherry cultivars distributed over the entire Campania territory were surveyed during years 2013–2016 to have a complete catalogue of the existing cultivars, recognize those threatened by the risk of extinction and characterize them at molecular/genetic level. Data were collected by specialized technicians who filled *ad hoc* forms during farmers’ interviews, explored available literature on the history of agriculture and election areas and, finally, reviewed all reported investigations carried out on the Campania Region genetic bank by other institutions. All accessions were additionally described and identified by morphological standard descriptors^51,52^. Fourteen additional sweet cherry accessions from different countries were obtained from the repository of the Fruit Tree Research Institute, Forlì, Italy and used as international references. The complete list of the cultivars investigated is shown in Table 1.

### Spatial data

Spatial data were organized and processed in a G.I.S. environment, using ESRI ArcGIS 10.2.2 software^53^. The geographic database included accessions’ descriptors and locations, climatic, geologic and pedologic data obtained by Italy phytoclimatic and geologic maps (Ministry of Environment and Protection of Land and Sea - General Direction for the Protection of Nature and the Sea) and Campania pedologic maps (Campania Region Geoportal - Territorial Information System of the Campania Region). Spatial overlay allowed to associate each accession to the pertaining bioclimate and soil composition. Accession’s spatial density was assessed using kernel function, specifically, the quartic kernel function described in Silverman^54^. Conceptually, each point, representing a single accession, was fitted with a smoothly curved surface with the highest value at the point location and diminishing values at increasing distance reaching zero at the search radius distance from the point. The search radius was defined using the default algorithm provided by the software. The output is a raster having a density at each cell calculated by adding the values of all the kernel surfaces where they overlay the cell centre.

## Supplementary information

Supplementary Information
