# Supplementary material for: Molecular and environmental analysis of Campania (Italy) sweet cherry (Prunus avium L.) cultivars for biocultural refugia identification and conservation
Source: Sci Rep. 2019 May 1;9:6796. doi: 10.1038/s41598-019-43226-2 (PMC6494815; doi:10.1038/s41598-019-43226-2)
Supplement: Supplementary file 1 — Supplementary Information [file 41598_2019_43226_MOESM1_ESM.pdf]

Molecular and environmental analysis of Campania (Italy) sweet cherry (*Prunus avium* L.) cultivars for biocultural refugia identification and conservation

Livio Muccillo, Vittorio Colantuoni, Rosaria Sciarrillo, Giuseppe Baiamonte, Giovanni Salerno, Mario Marziano, Lina Sabatino*, Carmine Guarino*

SUPPLEMENTARY FIGURES AND TABLES

Supplementary Figure S1

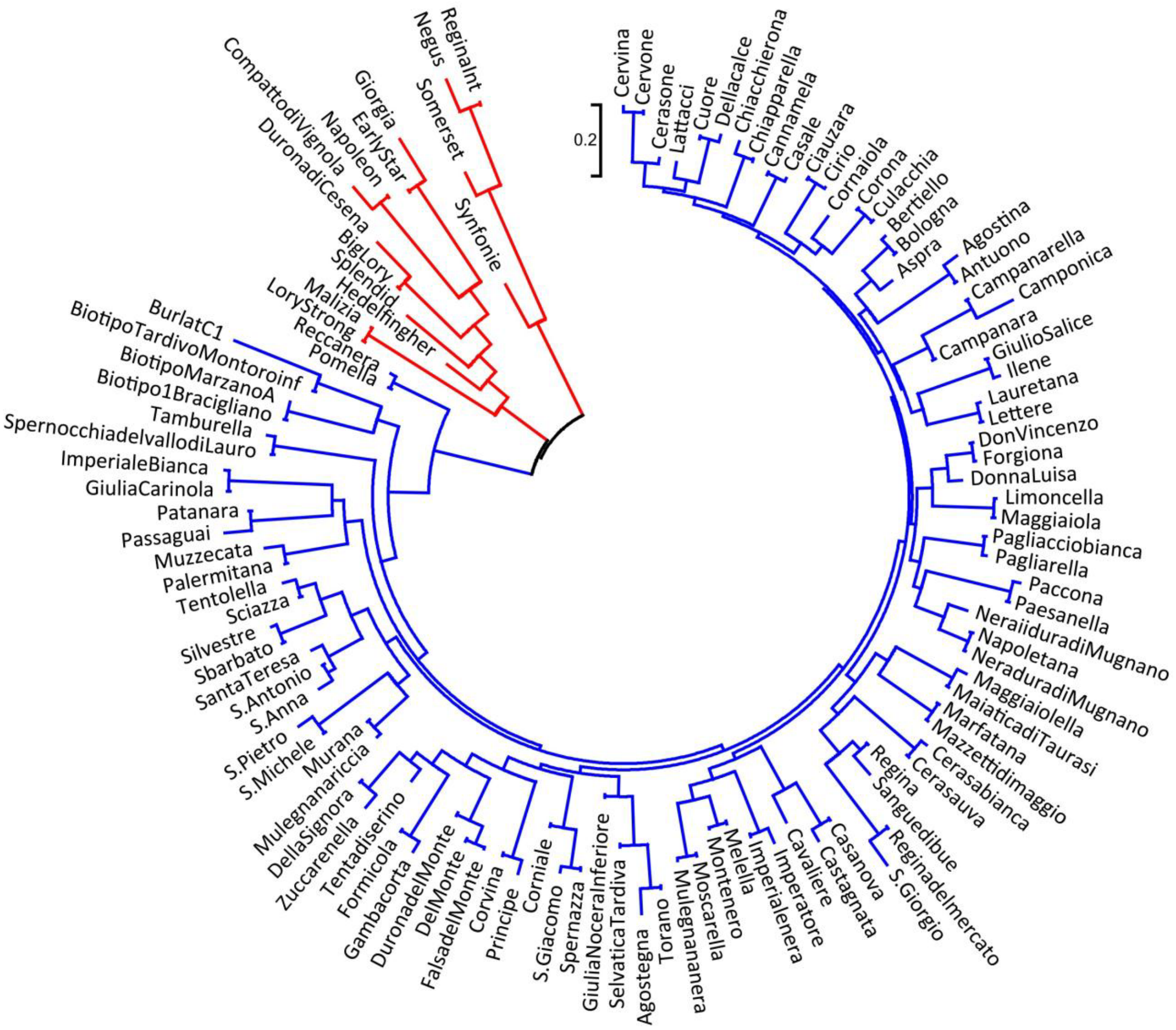

Supplementary Figure S1. Genetic relationships of Campania sweet cherry cultivars with International reference cultivars. The dendrogram was inferred using the the UPGMA method [56].

Supplementary Figure S2

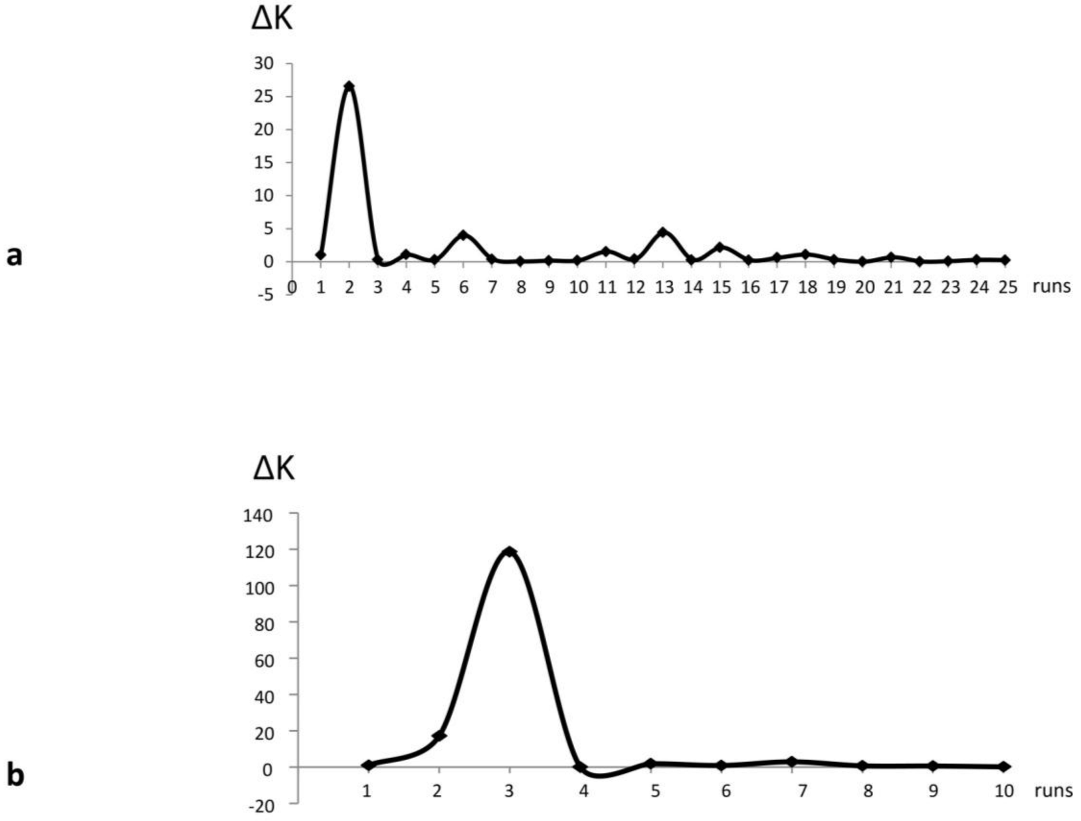

Supplementary Figure S2. Graph representation of the ΔK values obtained using STRUCTURE analysis.

(a) Best ΔK=2 was obtained with a burning period ranging from 5000 to 50000 with biased k from 1 to 25; **(b)** Best ΔK=3 was obtained with a burning period ranging from 500000 to 750000 with biased k from 1 to 10.

Supplementary Figure S3

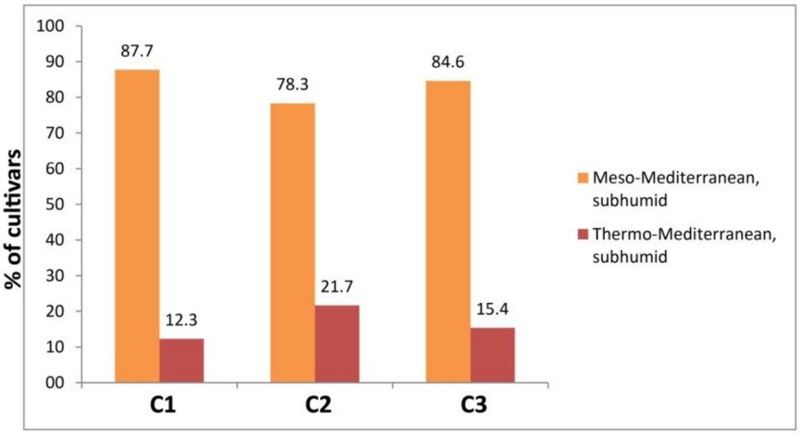

Supplementary Figure S3. Correlation of sweet cherry varieties' locations with bioclimate distribution.

83,5% of the analysed samples are exposed to the Meso-Mediterranean and the remaining 16,5% to the Thermo-Mediterranean bioclimate.

Supplementary Table S1

Locus	Accession No.	Repeat motif	Forward and Reverse primer sequences (5'-3')	Size range (bp)
EMPA001	AJ564644	$[AG]_4GGGT[AG]_{26}$	F: FAM-GCTCTGCTGCTTCAACCATT<br>R: TTCCCAACACATTACCCC	144-164
EMPA004	AJ564647	$[GA]_4AA[GA]_4AA[GA]_{15}$	F: NED-TACGGTAGGCTTCTGCAAGG<br>R: TTGGCAGCCAACAAAATCA	168-192
EMPA005	AJ564648	$[CT]_3CAT[CT]_{12}T[AC]_{23}$	F: PET-TGGGTTTGAGCAATATGCAA<br>R: CACCAATACACATGCACACG	160-264
EMPA011	AJ564654	$[AG]_{16}$	F: VIC-TGTGCTCACTCTCTGCTGCT<br>R: TGTGTGGGGTTCACAGTCTCC	222-256
EMPA018	AJ564661	$[AG]_{18}$	F: FAM-TCCAAGAACAAAGCCAAAATC<br>R: AATTTCAATGCATTCTGGATAG	79-111
EMPA015	AJ56458	$[GA]_{32}$	F: NED-TTTTGGTCAATCTGCTGCTG<br>R: CTCTCATCTTCCCCCTCCTC	193-252
EMPA012	AJ56455	$[AG]_{13}$	F: PET-TGTGTGCAAGGAAGCTTAAGAG<br>R: TGGCTGATTGGGGTTAAGTC	227-237
EMPAs06	ay526622	$[CT]_{12}$	F: VIC-AAGCGGAAAGCACAGGTAG<br>R: TTGCTAGCATAGAAAAGAATTGTAG	190-226
EMPAs02	ay526619	$[TTG]_7CTGC[TG]_{10}[AG]_8$	F: FAM-CTACTTCCATGATTGCCTCAC<br>R: AACATCCAGAACATCAACACAC	134-148
EMPAs10	ay526626	$[GA]_{28}$	F: NED-GCTAATATCAAATCCCAGCTCTC<br>R: TGAAGAAGTATGGCTTCTGTGG	154-191
EMPA016	AJ564659	$[AG]_{10}$	F: PET-CCAAACCCAATTACCCAGAG<br>R: GCCAATGTGCCAACCAATAAC	177-195
EMPAs13	ay526629	$[TC]_{11}[GT]_3AT[GT]_5$	F: VIC-GAATTGAAGCAACCAAGCAC<br>R: TGGCACACTCTACCTAACATTC	203-223
EMPAs12	ay526628	$[TG]_{10}A[GA]_{10}AA[GA]_{13}$	F: FAM-TGTGCTAATGCCAAAAATACC<br>R: ACATGCATTTCAACCCACTC	123-149
EMPAs01	ay526618	$[GA]_9[GA]_{11}$	F: PET-CAAAATCAACAAAATCTAAACC<br>R: CAAGAAGTCTCTAGCTCAAACC	224-242
EMPAs14	ay526630	$[TC]_{10}CCAT[TC]_5CCAT[TC]8$	F: VIC-TCCGCCATATCACAATCAAC<br>R: TTCCACACAAAAACCAATCC	173-212

Supplementary Table S1. List of the fifteen simple sequence repeats (SSRs) employed for genotyping the 113 cultivars, including nucleotide sequences, Accession No., repeat motifs and allelic size ranges (in bp).

Supplementary Table S2

ID	CV name	STRUCTURE nr	CV nr	EMPA001	EMPA001	EMPA004	EMPA004	EMPA005	EMPA005	EMPA011	EMPA011	EMPA012	EMPA012	EMPA015	EMPA015	EMPA016	EMPA016	EMPA018	EMPA018	EMPAs01	EMPAs01	EMPAs02	EMPAs02	EMPAs06	EMPAs06	EMPAs10	EMPAs10	EMPAs12	EMPAs12	EMPAs13	EMPAs13	EMPAs14	EMPAs14
CIL1CO1.1	Agostina	1	1	152	152	182	182	261	261	249	255	79	107	224	224	235	235	190	206	145	145	159	167	191	191	219	219	137	145	232	236	198	212
CIL1CO1.2	Agostina	2	1	152	152	182	182	261	261	249	255	79	107	224	224	235	235	190	206	145	145		167	177	191	219	219	137	145	232	236	198	212
CIL1CO1.3	Agostina	3	1	150	150	182	188	261	261	249	249	95	99	224	224	235	235	208	222	143	148	154	167	177	191	217	219		145	226	236	198	212
CIL2	Antuono	4	2	150	158	182	188	249	261	249	249	95	103	224	224	235	235	208	224	143	148	183	183	177	177	217	217	123	145	226	242	198	212
CIL3C2	Aspra	5	3	150	154	186	190	251	261	249	249	99	103	214	224	235	235	204	214	143	148	154	169	177	189	217	219	145	145	236	236	212	212
CIL3C3	Aspra	6	3	150	154	186	190	251	261	249	249	99	103	214	224	235	235	204	214	143	148	154	169	177	189	217	219	137	145	226	236	198	212
CIL3M1	Aspra	7	3	150	154	186	190	251	261	249	249	99	103	214	224	235	235	204	214	143	148	154	169	177	189	217	219	145	147	236	236	212	212
CIL3RM	Aspra	8	3	154	158	182	188	251	261	249	249	95	95	224	224	235	235	208	224	143	148	154	167	177	183	217	219	135	137	232	234	198	198
CIL4	Bertiello	9	4	150	158	188	188	251	261	249	249	79	99	224	224	235	235	190	208	143	148	154	183	177	177	217	217	145	147	226	236	212	212
CILBT1BR	Biotipo1Bracigliano	10	5	154	158	182	190	251	261	247	249	95	95	242	244	235	235	204	204	138	148	154	167	177	189	217	219	139	145	232	236	198	200
CILBT1	Biotipo1MarzanoA	11	6	150	150	188	188	160	261	249	249	95	99	214	226	235	235	222	224	141	143	154	167	177	191	219	219	145	147	226	236	212	212
CILBT2	Biotipo2MarzanoA	12	7	150	158	188	188	160	261	249	249	95	99	214	222	235	237	204	204	141	143	169	183	177	191	217	219	145	147	226	226	212	212
CILBT3	Biotipo3MarzanoA	13	8	150	158	188	188	251	261	249	249	79	95	222	222	235	235	204	208	141	145	169	183	177	191	217	217	143	145	232	236	198	212
CILBT4	Biotipo4MarzanoA	14	9	150	150	176	190	251	261	249	249	99	99	222	222	235	237	204	222	143	148	154	169	177	191	217	219	137	145	232	234	198	212
CILBTT	BiotipoTardivoMontoroinf	15	10	150	158	176	190	-9	-9	-9	-9	95	95	222	222	235	237	204	222	-9	-9	154	169	177	177	217	219	137	145	232	234	-9	-9
CIL6	Bologna	16	11	154	154	188	190	261	261	249	249	99	103	224	224	237	237	208	224	143	148	154	183	191	191	217	217	137	147	226	236	198	212
CIL8CV1	Campanara	17	12	154	158	188	190	251	261	249	249	95	99	224	228	235	235	208	224	143	148	167	183	177	191	217	219	123	145	226	236	198	212
CIL8S2(blu)	Campanara	18	12	148	148	182	188	261	261	249	249	95	95	224	224	235	235	204	208	148	148	167	183	177	191	217	219	137	147	226	236	198	212
CIL9	Campanarella	19	13	154	154	182	190	261	261	249	249	79	95	224	224	235	235	204	224	143	148	154	159	183	191	217	219	137	137	232	232	198	198
CIL9CH1	Campanarella	20	13	152	154	182	188	251	261	249	249	95	99	224	228	235	237	204	208	145	145	183	183	191	191	217	217	145	145	226	232	198	198
CIL10	Camponica	21	14	154	154	188	190	-9	-9	-9	-9	79	103	224	224	235	235	208	224	148	148	154	167	177	191	217	219	145	145	236	236	198	212
CIL11	Cannamela	22	15	150	150	188	188	251	261	249	249	95	99	224	224	235	235	204	208	141	148	183	183	177	191	217	217	145	145	226	236	212	212
CIL14	Casale	23	16	150	158	188	190	251	261	249	249	95	99	214	224	235	237	204	220	143	148	154	169	177	191	219	219	145	147	226	236	212	212
CIL15S	Casanova	24	17	144	150	182	188	251	261	-9	-9	-9	-9	224	230	-9	-9	-9	-9	-9	-9	167	167	-9	-9	219	219	145	145	-9	-9	198	212
CIL16NI1	Castagnata	25	18	-9	-9	182	182	261	261	249	249	79	107	-9	-9	235	235	204	208	141	148	169	183	-9	-9	217	217	137	145	226	226	198	212
CIL18G	Cavaliere	26	19	150	154	188	190	246	261	249	249	95	99	224	244	237	237	204	208	141	148	159	183	177	191	217	217	145	145	226	232	198	212
CIL19	Cerasabianca	27	20	150	158	176	186	-9	-9	249	249	95	107	224	252	235	235	208	224	145	148	154	179	177	191	217	217	137	145	232	234	198	212
CIL21A1(blu)	Cerasauva	28	21	150	150	188	190	251	261	249	255	79	103	228	228	235	235	224	224	148	148	167	167	177	191	217	219	137	137	226	236	198	212
CIL21RM1	Cerasauva	29	21	144	154	188	190	251	261	249	253	95	99	224	244	237	237	202	206	141	148	154	159	183	191	217	219	137	145	232	242	198	198
CIL22CM1	Cerasone	30	22	150	150	182	190	251	261	249	249	99	99	224	224	235	235	204	214	143	148	154	169	177	191	217	219	145	147	226	236	212	212
CIL22G	Cerasone	31	22	150	158	188	188	259	261	249	249	95	99	214	224	235	235	204	220	143	148	154	167	177	191	217	219	145	147	226	236	212	212
CIL23	Cervina	32	23	148	150	188	188	261	261	249	255	95	99	224	224	235	237	202	224	143	148	167	183	191	191	217	217	137	147	226	236	212	212
CIL24	Cervone	33	24	150	150	188	190	259	259	249	249	95	99	224	224	235	235	208	220	143	143	154	154	191	191	217	219	145	145	226	236	212	212
CIL24.1	Cervone	34	24	150	150	188	190	259	259	249	249	95	99	224	224	235	235	208	220	143	143	154	154	177	191	217	219	145	145	226	236	212	212
CIL25S1(nero)	Chiacchierona	35	25	144	150	188	188	251	251	249	249	95	99	224	224	237	237	202	220	141	148	154	183	177	191	217	219	137	145	226	226	198	198
CIL25S3(nero)	Chiacchierona	36	25	144	150	188	188	261	261	249	249	95	99	224	224	235	235	202	220	141	148	154	183	191	191	217	219	137	145	226	226	198	198
CIL26SADG1	Chiapparella	37	26	150	150	188	188	261	261	249	249	95	99	224	228	237	237	206	208	143	143	156	169	177	191	217	217	145	145	226	242	198	198
CIL26SADG2	Chiapparella	38	26	150	158	188	190	261	261	249	249	95	99	214	222	235	237	204	222	143	148	154	169	177	191	219	219	145	147	226	236	212	212
CIL27	Ciauzara	39	27	150	154	182	190	261	261	249	249	95	103	214	224	235	235	202	208	148	148	154	154	191	191	217	219	137	145	232	236	198	198
CIL28S1(nero)	Cirio	40	28	150	158	188	190	251	261	249	249	95	99	224	224	235	235	202	208	141	141	167	183	191	191	217	219	145	147	236	236	198	212
CIL29	Cornaiola	41	29	154	154	188	190	261	261	249	249	95	99	224	228	235	235	220	224	143	148	167	183	177	191	219	219	145	147	232	236	198	212
CIL29C1a	Cornaiola	42	29	144	154	182	188	249	251	249	249	99	99	224	230	235	235	204	204	141	148	167	183	177	191	217	219	145	145	226	234	198	212
CIL29M1	Cornaiola	43	29	154	154	188	190	261	261	249	249	-9	-9	224	228	235	237	220	224	143	148	167	183	177	191	217	217	145	149	226	236	198	212
CIL110MA3	Corniale	44	30	154	158	188	188	247	261	249	249	95	103	222	222	235	237	208	226	141	145	154	167	189	191	217	217	139	145	232	234	198	212
CIL30G	Corona	45	31	150	158	188	190	251	251	249	249	95	103	224	224	235	235	220	220	143	148	154	167	191	191	217	217	137	145	226	236	198	212
CIL100SM1nuovo	Corvina	46	32	150	158	182	182	247	261	249	249	95	99	222	222	235	237	208	224	143	148	154	183	189	191	217	217	145	147	226	236	198	212
CIL31	Culacchia	47	33	150	150	176	190	261	261	249	249	99	107	224	224	235	235	220	220	143	148	154	179	177	191	217	219	139	147	232	234	212	212
CIL31CM1	Culacchia	48	33	154	158	182	190	251	261	249	249	95	95	224	224	237	237	208	220	135	148	154	154	177	191	219	219	137	145	232	236	198	212
CIL31SST1	Culacchia	49	33	150	150	176	190	261	261	249	249	99	107	224	224	235	235	220	220	143	148	154	179	177	191	217	219	139	147	232	234	212	212
CIL32SST1	Cuore	50	34	150	150	188	190	261	261	249	249	95	99	224	224	237	237	208	224	138	143	159	183	177	191	217	219	137	145	226	236	212	212
CIL33	Dellacalce	51	35	150	158	188	190	261	261	222	249	95	99	214	224	235	235	202	220	143	148	154	169	177	191	219	219	145	147	226	236	212	212
CIL107SM2	DelMonte	52	36	150	158	188	188	247	261	249	249	95	99	222	222	235	237	208	224	145	148	169	183	177	189	217	217	143	145	226	236	212	212
CIL35MT5	DonnaLuisa	53	37	158	158	188	188	251	261	249	249	95	99	224	224	237	237	208	220	143	148	169	183	177	191	217	217	145	145	226	236	212	212
CIL35S1	DonnaLuisa	54	37	148	150	182	188	261	261	249	249	95	99	224	224	235	235	208	224	143	148	154	169	191	191	217	219	145	147	226	236	198	212
CIL36	DonVincenzo	55	38	150	154	188	188	261	261	249	249	95	95	224	224	235	235	208	208	141	143	183	183	177	191	217	217	137	145	226	232	198	198
CIL105SM1	DuronadelMonte	56	39	150	158	188	188	247	261	249	249	95	99	222	222	235	237	208	208	145	148	169	183	177	189	217	217	143	145	226	236	212	212
CIL106SM1	FalsadelMonte	57	40	150	158	188	188	247	261	249	249	95	99	222	222	235	237	208	224	145	148	169	183	177	189	217	217	143	145	226	236	212	212
CIL37MA1	Forgiona	58	41	150	150	188	188	261	261	249	249	95	99	214	228	235	235	220	220	141	143	154	167	177	191	217	217	145	147	226	236	212	212
CIL37SADG1	Forgiona	59	41	150	154	188	190	251	251	249	249	95	103	224	224	235	235	220	220	145	148	154	167	189	191	217	219	137	145	226	236	198	212
CIL104MA1	Formicola	60	42	150	158	188	188	261	261	249	249	95	99	214	222	235	237	204	204	141	143	169	183	177	191	217	219	145	147	226	226	212	212
CIL104MA1.2	Formicola	61	42	150	154	188	188	261	261	249	249	79	100	222	222	235	237	208	224	141	145	154	183	177	189	217	217	137	145	226	232	198	212
CIL103FA1	Gambacorta	62	43	154	154	182	188	247	261	249	249	95	99	244	244	235	237	208	208	135	143	154	187	189	191	217	219	137	147	236	242	198	200
CIL103MT1	Gambacorta	63	43	154	158	182	188	247	261	249	249	97	99	222	244	237	237	208	208	141	143	154	169	177	191	217	217	123	147	232	234	198	198
CIL103MT2nuovo	Gambacorta	64	43	154	158	182	188	247	261	249	249	97	99	222	244	237	237	208	208	141	143	154	169	177	191	217	217	123	147	232	234	198	198
CIL115C1	GiuliaCarinola	65	44	144	150	188	188	251	251	249	249	99	99	230	230	235	235	204	204	143	148	167	183	189	191	219	219	145	145	226	242	198	198
CIL115NI	GiuliaNoceraInferiore	66	45	144	150	182	182	-9	-9	222	249	95	99	222	224	235	237	204	204	138	143	154	169	177	191	217	219	145	147	-9	-9	198	200
CIL38M1	GiulioSalice	67	46	150	154	182	182	259	259	249	249	79	99	214	224	235	235	204	208	138	148	159	183	177	191	217	219	137	139	232	236	198	212
CIL39G	Ilene	68	47	144	154	182	188	249	251	249	249	99	99	224	230	235	235	204	204	141	148	167	183	177	191	217	219	145	145	226	232	198	212
CIL39MA1	Ilene	69	47	144	154	182	188	249	251	249	249	99	99	224	230	235	235	204	204	141	148	167	183	177	191	217	219	145	145	226	232	198	212
CIL40	Imperatore	70	48	150	150	182	188	251	261	249	249	95	99	-9	-9	237	237	208	208	143	148	169	183	191	191	217	217	145	147	226	232	212	212
CIL116	ImperialeBianca	71	49	150	158	188	188	261	261	249	249	95	99	214	224	235	237	204	204	141	143	169	183	177	191	217	219	145	147	226	226	212	212
CIL41CM1	Imperialenera	72	50	150	150	182	190	251	261	249	249	99	99	214	224	235	237	204	214	145	148	154	169	177	191	217	219	145	147	226	236	198	212
CIL41FA1	Imperialenera	73	50	150	150	176	190	261	261	249	249	99	107	224	224	235	235	220	220	143	148	154	179	177	191	217	219	139	147	232	234	212	212
CIL41M1	Imperialenera	74	50	150	158	188	190	261	261	249	249	95	99	214	224	235	237	204	220	143	148	154	169	177	191	219	219	145	147	226	236	198	198
CIL41S1(blu)	Imperialenera	75	50	150	158	188	190	261	261	249	249	95	99	214	224	235	237	204	220	143	148	154	169	177	191	219	219	145	147	226	236	212	212
CIL42	Lattacci	76	51	150	158	182	188	261	261	249	249	95	95	224	224	235	235	208	208	141	148	154	183	191	191	217	217	145	145	232	236	198	212
CIL42S1	Lattacci	77	51	154	158	182	188	247	261	249	251	95	95	224	224	235	235	208	220	141	148	154	154	177	191	217	219	139	145	232	236	212	212
CIL43	Lauretana	78	52	144	154	182	188	249	251	249	249	99	99	224	230	235	235	204	204	141	148	167	183	177	191	217	219	145	145	226	232	198	212
CIL44SA2	Lettere	79	53	150	154	188	188	261	261	249	249	95	99	224	224	235	235	208	220	141	143	169	183	177	191	217	219	123	145	226	236	198	212
CIL45	Limoncella	80	54	158	164	188	190	261	261	249	249	95	95	224	244	235	235	206	208	138	141	154	154	177	191	217	217	139	139	234	236	198	212
CIL46C3	Maggiaiola	81	55	150	150	182	188	251	261	249	249	99	109	224	224	235	235	208	224	143	145	167	183	177	189	217	219	123	137	232	242	198	212
CIL46MA1nuovo	Maggiaiola	82	55	154	154	182	188	247	261	249	249	95	99	244	244	235	235	208	208	135	143	154	154	191	191	217	219	137	147	236	242	198	198
CIL47	Maggiaiolella	83	56	150	158	190	190	251	261	249	249	95	95	224	224	237	237	208	220	135	148	154	167	177	191	217	219	137	145	232	232	198	212
CIL48BR1nuovo	MaiaticadiTaurasi	84	57	154	158	182	188	247	261	249	251	95	95	224	224	235	235	208	220	138	148	154	167	-9	-9	217	219	139	145	232	236	198	198
CIL48MA1	MaiaticadiTaurasi	85	57	154	154	182	190	261	261	249	256	79	95	-9	-9	235	235	214	224	145	148	154	167	191	191	217	219	137	145	232	242	198	198
CIL48S1(nero)	MaiaticadiTaurasi	86	57	154	154	182	190	251	261	249	249	95	99	244	244	-9	-9	208	208	135	143	154	154	191	191	217	219	137	147	236	242	198	198
CIL48S4	MaiaticadiTaurasi	87	57	154	158	188	188	251	261	249	249	95	99	214	224	235	237	204	224	145	148	154	167	191	191	217	219	137	139	236	236	212	212
CIL49	Marfatana	88	58	150	150	182	190	251	261	249	249	79	111	214	224	235	235	204	204	145	148	167	167	177	191	217	219	123	147	232	242	198	198
CIL50(nero)	Mazzettidimaggio	89	59	150	150	182	182	259	259	249	249	95	99	224	224	235	237	204	208	138	143	154	167	177	191	217	219	137	147	226	232	198	198
CIL50MA1	Mazzettidimaggio	90	59	150	150	182	190	251	261	249	249	97	97	214	224	235	237	204	214	145	148	154	169	177	191	217	219	145	147	226	236	212	212
CIL50SO1	Mazzettidimaggio	91	59	150	150	182	190	251	261	249	249	99	99	214	224	235	237	204	214	145	148	154	169	177	191	217	219	145	147	226	236	212	212
CIL51G	Melella	92	60	150	150	188	188	261	261	249	249	95	99	214	228	235	235	220	220	141	143	154	167	177	191	219	219	145	147	226	236	212	212
CIL51MA1nuovo	Melella	93	60	150	150	188	188	261	261	249	249	95	99	214	228	235	235	222	224	141	143	154	167	177	191	219	219	145	147	226	236	212	212
CIL51MA2nuovo	Melella	94	60	150	158	176	182	251	251	249	249	93	93	-9	-9	235	237	222	224	141	148	154	159	191	191	217	219	137	139	236	236	198	212
CIL52	Montenero	95	61	144	150	188	190	251	261	249	249	79	95	-9	-9	235	235	204	224	143	145	167	183	177	191	217	219	145	147	226	232	198	212
CIL53MA1	Moscarella	96	62	150	158	188	188	261	261	249	249	93	95	-9	-9	235	237	204	204	141	143	169	183	177	191	217	219	145	147	226	226	212	212
CIL54	Mulegnananera	97	63	150	154	188	190	261	261	249	249	93	99	-9	-9	235	237	208	224	143	145	167	167	177	191	217	217	145	145	226	232	198	198
CIL55	Mulegnanariccia	98	64	150	154	188	190	261	261	249	249	95	99	222	222	235	235	204	208	141	143	183	183	177	177	217	219	145	147	226	236	198	212
CIL55CH1	Mulegnanariccia	99	64	150	154	188	190	261	261	249	249	95	99	222	222	237	237	204	208	141	143	183	183	177	177	217	219	145	147	226	236	198	212
CIL55M1	Mulegnanariccia	100	64	150	154	188	190	261	261	249	249	95	99	222	222	235	235	204	208	141	143	183	183	177	177	217	219	145	147	226	236	198	212
CIL56	Murana	101	65	150	158	188	190	251	261	249	249	95	99	222	222	235	235	208	220	141	143	154	169	177	191	217	219	145	147	226	236	198	198
CIL119SADG1	Muzzecata	102	66	150	158	188	190	261	261	249	249	79	79	228	228	235	235	224	224	145	148	154	167	189	191	219	219	137	145	232	234	198	212
CIL57G	Napoletana	103	67	154	158	182	190	251	261	249	249	95	95	222	222	235	235	204	220	135	148	154	154	177	191	219	219	137	145	232	236	198	212
CIL58	NeraduradiMugnano	104	68	150	158	182	188	251	261	249	249	95	99	222	234	235	235	204	220	143	148	154	169	177	191	217	217	137	145	232	236	198	212
CIL59SM1	NeraiiduradiMugnano	105	69	150	150	188	190	261	261	249	249	95	99	222	222	233	235	208	222	141	143	167	183	191	191	217	219	123	145	236	236	198	198
CIL60	Paccona	106	70	154	158	188	190	251	263	249	249	95	99	224	224	233	233	208	222	141	148	154	167	177	191	217	219	145	145	232	236	198	212
CIL61	Paesanella	107	71	144	150	188	188	261	261	249	249	95	99	222	222	235	235	208	224	145	148	169	169	177	191	217	217	-9	-9	232	236	212	212
CIL62	Pagliacciobianca	108	72	150	158	182	188	251	261	249	249	95	99	222	234	235	235	204	222	143	148	154	169	177	191	217	217	137	145	226	236	198	212
CIL62BR1.1	Pagliacciobianca	109	72	154	158	182	190	251	261	249	249	95	95	244	244	235	235	202	202	138	148	154	167	177	191	217	219	139	145	232	236	198	198
CIL62BR1.2	Pagliacciobianca	110	72	150	154	182	182	251	261	249	249	79	95	224	226	235	237	206	208	138	143	154	167	177	191	217	219	137	137	232	232	198	212
CIL62BR2	Pagliacciobianca	111	72	150	158	182	188	251	261	249	249	95	99	224	236	237	237	204	208	143	148	154	169	177	191	217	217	137	145	226	236	198	212
CIL62MT1nuovo	Pagliacciobianca	112	72	150	158	182	188	251	261	249	249	95	99	222	234	235	235	204	222	143	148	154	169	177	191	217	217	137	145	226	236	198	212
CIL63	Pagliarella	113	73	-9	-9	188	192	-9	-9	-9	-9	95	95	214	214	235	237	204	208	143	145	154	169	177	177	217	219	145	145	226	236	198	212
CIL117NI1	Palermitana	114	74	150	150	182	182	251	251	249	249	95	99	224	224	235	237	204	208	138	143	154	167	177	189	217	219	137	147	226	232	198	198
CIL67	Passaguai	115	75	150	150	182	182	259	259	249	249	95	99	224	224	235	237	204	208	138	143	154	167	177	191	217	219	137	147	226	232	198	198
CIL68	Patanara	116	76	150	150	182	188	261	261	249	249	95	99	222	222	235	235	204	208	143	148	167	167	191	191	217	219	145	145	236	236	198	212
CIL71	Pomella	117	77	-9	-9	-9	-9	251	261	-9	-9	79	107	-9	-9	-9	-9	208	222	138	148	154	154	177	191	217	219	-9	-9	232	236	198	212
CIL99BR1	Principe	118	78	150	154	182	190	247	259	249	249	95	95	214	224	235	235	208	224	138	143	154	167	177	195	217	219	137	147	226	236	198	212
CIL73C2	Reccanera	119	79	150	150	182	188	-9	-9	-9	-9	79	95	214	222	235	235	204	208	143	148	169	183	177	183	217	219	123	147	226	232	198	212
CIL73CH1(blu)	Reccanera	120	79	150	150	176	190	251	261	249	249	99	99	222	222	235	237	204	222	143	148	154	169	-9	-9	217	219	137	145	232	234	212	212
CIL73MA1	Reccanera	121	79	164	164	182	182	261	261	249	254	95	99	218	222	235	235	206	222	138	138	154	154	191	191	217	219	145	145	232	232	198	212
CIL73RM1	Reccanera	122	79	164	164	182	182	261	261	249	254	95	99	218	222	235	235	206	222	138	138	154	154	189	191	217	219	145	145	232	232	198	212
CIL73SA1(nero)	Reccanera	123	79	150	154	188	188	261	261	249	249	95	99	222	222	235	235	208	222	143	143	154	183	177	191	217	219	145	147	226	232	198	212
CIL75.1	Regina	124	80	150	158	176	186	247	261	249	249	95	107	224	224	235	235	208	222	145	148	154	179	177	191	217	217	139	145	232	234	198	212
CIL75.2	Regina	125	80	150	158	176	186	247	261	249	249	95	107	224	224	235	235	208	224	145	148	154	179	177	191	217	217	139	145	232	234	198	212
CIL75G	Regina	126	80	154	158	182	190	251	251	249	249	95	107	244	252	235	235	204	222	138	143	167	167	177	189	217	219	137	145	232	236	198	198
CIL76G	Reginadelmercato	127	81	150	154	168	186	247	261	232	249	79	95	193	197	229	235	204	218	135	145	154	169	177	189	217	219	137	145	226	232	173	198
CIL109MA1	S.Giacomo	128	82	148	158	188	188	247	261	249	249	95	107	214	226	235	237	204	224	141	143	183	191	189	191	219	219	143	145	226	236	198	212
CIL109MA1nuovo	S.Giacomo	129	82	150	158	188	188	247	261	249	249	95	107	214	226	235	237	204	224	141	143	183	191	177	191	219	219	143	145	226	236	198	212
CIL109MA4	S.Giacomo	130	82	150	158	182	188	251	261	249	249	79	107	224	224	235	235	206	208	141	148	154	159	177	191	217	219	137	139	232	236	198	198
CIL78(1)	S.Giorgio	131	83	150	158	176	186	247	261	249	249	95	107	224	224	235	235	208	224	145	148	154	179	177	191	217	217	139	145	232	234	198	212
CIL82(1)	S.Michele	132	84	144	150	182	188	251	259	249	249	95	99	222	222	235	237	204	224	145	148	154	167	177	189	217	217	137	147	226	226	198	198
CIL83RM1nuovo	S.Pietro	133	85	154	158	182	190	261	261	249	249	79	95	224	226	235	237	206	222	143	148	154	167	177	191	219	219	123	137	232	236	198	198
CIL79MT1	Sanguedibue	134	86	150	150	188	188	261	261	249	249	95	99	222	222	235	237	208	224	143	143	167	183	189	191	217	219	123	145	236	236	198	198
CIL85(1)	SantAnna	135	87	150	158	188	188	261	261	249	249	95	99	214	222	235	237	204	222	143	148	154	169	177	191	219	219	145	147	226	236	212	212
CIL85NI1	SantAnna	136	87	-9	-9	182	188	251	261	249	249	95	95	222	222	237	237	208	208	143	148	169	183	189	191	217	217	145	147	226	232	212	212
CIL86(blu)	SantAntonio	137	88	150	158	188	188	261	261	249	249	95	99	214	222	235	237	204	222	143	148	154	169	177	191	219	219	145	147	226	236	212	212
CIL86G	SantAntonio	138	88	154	158	182	190	247	261	249	249	95	95	222	222	237	237	208	222	135	148	154	154	177	189	219	219	137	145	226	236	198	212
CIL87(1)	SantaTeresa	139	89	150	150	188	188	261	261	249	249	95	99	222	222	235	237	208	222	143	143	167	183	191	191	217	219	123	145	236	236	198	198
CIL88	Sbarbato	140	90	150	150	182	188	251	261	249	249	95	99	222	222	237	237	208	208	143	148	169	183	189	191	217	217	145	147	226	232	212	212
CIL101BR1	Sciazza	141	91	144	150	182	190	251	259	249	249	95	99	222	222	235	237	204	208	138	148	167	167	189	191	217	217	137	147	226	236	198	212
CIL101MT2(nero)	Sciazza	142	91	154	158	182	188	251	261	249	249	97	99	222	244	237	237	208	208	141	143	154	169	177	191	217	217	123	147	232	234	198	198
CIL89	Silvestre	143	92	150	150	182	188	251	261	249	249	95	99	222	222	237	237	208	208	143	148	169	183	189	191	217	217	145	147	226	232	212	212
CIL108C3nuovo	Spernazza	144	93	150	158	188	188	247	261	249	249	95	107	214	226	235	237	204	224	141	143	183	191	189	191	219	219	143	145	226	236	198	212
CIL91BR1	SpernocchiadelvallodiLauro	145	94	154	158	182	190	247	261	249	249	95	95	244	244	235	235	204	204	138	148	154	167	177	189	217	219	139	145	232	236	198	200
CIL91MT1(blu)	SpernocchiadelvallodiLauro	146	94	154	158	182	190	247	261	249	249	95	95	244	244	235	235	204	204	138	148	154	167	177	189	217	219	139	145	232	236	198	200
CIL91MT3	SpernocchiadelvallodiLauro	147	94	154	158	182	190	247	261	249	249	95	95	244	244	235	235	204	204	138	148	154	167	177	189	217	219	139	145	232	236	198	200
CIL91NI1	SpernocchiadelvallodiLauro	148	94	154	158	182	190	247	261	249	249	95	95	244	244	235	235	204	204	138	148	154	167	177	189	217	219	139	145	232	236	198	200
CIL93	Tamburella	149	95	150	150	182	190	251	261	249	249	99	99	214	224	235	237	204	214	145	148	154	169	177	191	217	219	145	147	226	236	212	212
CIL93(blu)	Tamburella	150	95	150	150	182	190	251	261	249	249	99	99	214	224	235	237	204	214	145	148	154	169	177	191	217	219	145	147	226	236	212	212
CIL94S1(blu)	Tentadiserino	151	96	154	158	188	188	247	247	249	249	99	103	222	222	235	235	204	224	143	145	154	183	189	191	217	217	137	147	226	236	198	198
CIL94S4(blu)	Tentadiserino	152	96	154	158	188	188	247	247	249	249	99	103	222	222	235	235	204	224	143	145	154	183	191	191	217	217	137	147	226	236	198	198
CIL102S2(nero)	Tentolella	153	97	154	158	188	188	247	247	249	249	99	103	222	222	235	235	204	224	143	145	154	183	189	191	217	217	137	147	226	236	198	198
CIL111MA3	Torano	154	98	152	154	182	182	261	261	249	254	79	107	222	222	235	235	206	206	141	145	154	183	177	191	217	217	137	145	232	236	198	212
CIL97	Zuccarenella	155	99	150	154	182	188	242	261	249	249	99	103	222	222	235	235	208	224	143	145	154	183	177	191	217	217	123	145	232	232	198	212

Supplementary Table S2. List of the Campania's sweet cherry accessions analysed reporting the individual identification code (ID), related cultivar name (CV name), STRUCTURE individual number (STRUCTURE nr), cultivar number (CV nr) and all the amplified alleles of the SSRs loci as reported in Supplementary Table S1.

Supplementary Table S3

Locus	k	N	HExp	HObs	PIC	PD	P. A.	ARR	F(Null)	Fis	Fst	Fit
EMPA001	7	200	0,684	0,635	0,635	0,854	0	ND	0,037	-0.719	0.462	0.076
EMPA004	7	204	0,687	0,657	0,628	0,753	2	0,286	0,028	-0.716	0.494	0.131
EMPA005	13	194	0,62	0,552	0,581	0,662	5	0,385	0,045	-0.709	0.580	0.283
EMPA011	10	197	0,185	0,107	0,180	0,412	4	0,400	0,260	-0.796	0.821	0.678
EMPA018	12	203	0,694	0,764	0,642	0,789	4	0,333	-0,056	-0.786	0.393	-0.084
EMPA015	19	196	0,747	0,408	0,784	0,861	6	0,315	0,307	-0.543	0.684	0.512
EMPA012	6	196	0,497	0,337	0,427	0,552	2	0,333	0,187	-0.587	0.634	0.419
EMPAs06	12	205	0,81	0,737	0,710	0,711	1	0,083	0,042	-0.713	0.477	0.104
EMPAs02	7	202	0,774	0,871	0,737	0,88	0	ND	-0,069	-0.806	0.418	-0.050
EMPAs10	10	205	0,748	0,785	0,710	0,954	3	0,300	-0,027	-0.765	0.439	0.010
EMPA016	5	201	0,615	0,801	0,534	0,662	1	0,200	-0,140	-0.873	0.350	-0.218
EMPAs13	7	206	0,538	0,583	0,438	0,623	1	0,143	-0,045	-0.817	0.458	0.016
EMPAs12	10	203	0,715	0,828	0,672	0,845	2	0,200	-0,083	-0.790	0.406	-0.063
EMPAs01	9	197	0,736	0,843	0,687	0,722	3	0,333	-0,077	-0.806	0.473	0.049
EMPAs14	4	202	0,53	0,465	0,417	0,556	1	0,250	0,064	-0.669	0.520	0.198
Mean	9,20	200,7	0,639	0,625	0,585	0,722	0.233	0,238	0,032	-0.740	0.507	0.137

Supplementary Table S3. Statistical analysis' data obtained by genotyping both Campania's and International reference's 206 sweet cherry individuals corresponding to 113 cultivars, using the fifteen SSR microsatellite markers.

For each locus, the allele number (k), the number of amplified samples (N), the expected (He) and observed (Ho) heterozygosity, the Polymorphic Index Content (PIC), Power Discrimination (PD), the private (P.A.)/total Alleles Richness Ratio (ARR), null alleles frequencies, F (Null), Inbreeding coefficient (Fis), Fixation Index (Fst) and Fit (Fitness) were calculated.
